# Simulation of Varroa mite control in honey bee colonies without synthetic acaricides: Demonstration of Good Beekeeping Practice for Germany in the BEEHAVE model

**DOI:** 10.1002/ece3.9456

**Published:** 2022-11-08

**Authors:** Isabel Schödl, Richard Odemer, Matthias A. Becher, Stefan Berg, Christoph Otten, Volker Grimm, Jürgen Groeneveld

**Affiliations:** ^1^ Department of Ecological Modelling Helmholtz Centre for Environmental Research – UFZ Leipzig Germany; ^2^ Julius Kühn‐Institute (JKI), Federal Research Centre for Cultivated Plants Institute for Bee Protection Braunschweig Germany; ^3^ Artificial Life Laboratory, Institute of Biology, Karl‐Franzens University Graz Graz Austria; ^4^ Bavarian State Institute for Viticulture and Horticulture, Institute for Bee Research and Beekeeping Veitshöchheim Germany; ^5^ Service Centre for Rural Areas (DLR), Expert Centre for Bees and Beekeeping Mayen Germany; ^6^ Plant Ecology and Nature Conservation University of Potsdam Potsdam Germany

**Keywords:** acaricides, BEEHAVE, beekeeping, drones, education, Honey bees, modelling, pest control, varroa mite

## Abstract

The BEEHAVE model simulates the population dynamics and foraging activity of a single honey bee colony (*Apis mellifera*) in great detail. Although it still makes numerous simplifying assumptions, it appears to capture a wide range of empirical observations. It could, therefore, in principle, also be used as a tool in beekeeper education, as it allows the implementation and comparison of different management options. Here, we focus on treatments aimed at controlling the mite *Varroa destructor*. However, since BEEHAVE was developed in the UK, mite treatment includes the use of a synthetic acaricide, which is not part of Good Beekeeping Practice in Germany. A practice that consists of drone brood removal from April to June, treatment with formic acid in August/September, and treatment with oxalic acid in November/December. We implemented these measures, focusing on the timing, frequency, and spacing between drone brood removals. The effect of drone brood removal and acid treatment, individually or in combination, on a mite‐infested colony was examined. We quantify the efficacy of Varroa mite control as the reduction of mites in treated bee colonies compared to untreated bee colonies. We found that drone brood removal was very effective, reducing mites by 90% at the end of the first simulation year after the introduction of mites. This value was significantly higher than the 50–67% reduction expected by bee experts and confirmed by empirical studies. However, literature reports varying percent reductions in mite numbers from 10 to 85% after drone brood removal. The discrepancy between model results, empirical data, and expert estimates indicate that these three sources should be reviewed and refined, as all are based on simplifying assumptions. These results and the adaptation of BEEHAVE to the Good Beekeeping Practice are a decisive step forward for the future use of BEEHAVE in beekeeper education in Germany and anywhere where organic acids and drone brood removal are utilized.

## Graphical abstract

The honey bee model BEEHAVE has been used to simulate different management options to control the Varroa mite that is a major cause of colony failure. The simulations confirmed the high efficacy of good beekeeping practice suggested by German authorities. However, the simulated efficacy of drone brood removal seems to be too high, which is a plea for further research in the details of this particular measure.

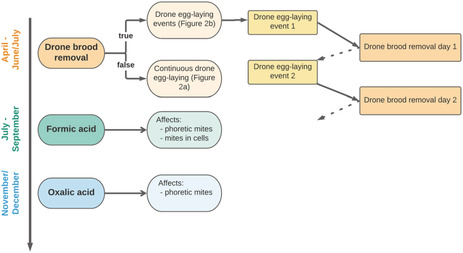

## 1 INTRODUCTION

A major threat to the Western honey bee, *Apis mellifera* L., and to global apiculture, is the ectoparasitic mite *Varroa destructor* (hereinafter referred to as varroa, mite, or varroa mite) (Rosenkranz et al., 2010). It affects bee colonies predominantly through the transmission of viruses, e.g., deformed wing virus (DWV) or acute bee paralysis virus (ABPV) (Carreck et al., 2010; Govan et al., 2000; Lanzi et al., 2006). A bee colony not being treated against varroa will usually die within one to three years (Fries et al., 2006; Martin, 1998). Beekeepers and researchers therefore established varroa control strategies to ensure the health and survival of honey bee colonies (Aumeier et al., 2012; LWG, 2020; MLR BW, 2020; van der Steen & Vejsnæs, 2021).

Treatment with synthetic chemical acaricides, such as pyrethroids, amidines, or organophosphates, are widely used by beekeepers due to their convenience. However, a major downside of these pesticides is that mite populations can become resistant to regular use (Kanga et al., 2016; Martin, 2004; Rinkevich, 2020). This ultimately requires increasing doses or alternating the application of different compounds. As a result, these substances accumulate in beeswax and can cause residues in honey and other bee products at levels that may even affect larvae and adults (Dai et al., 2018; Mullin et al., 2010).

In Germany, beekeepers are therefore very critical of the use of synthetic pesticides in beekeeping. Instead, biological mite control has been introduced as part of a strategy of Good Beekeeping Practice. Drone brood removal (DBR) is a cornerstone of this strategy and is carried out from April to June to reduce mite numbers during summer. It is an effective measure to reduce mite levels since varroa prefers to infest drone brood cells (Boot et al., 1995; Issa et al., 1993; van der Steen & Vejsnæs, 2021). At the end of the season, organic acids are used to control mites and prepare colonies for overwintering (LWG, 2020; MLR BW, 2020). These acids are natural components of honey and do not produce residues in hive products when applied after harvest (Bogdanov et al., 2002).

Some bee institutes, authorities and beekeepers' associations in Germany provide tools to facilitate the management of the varroa mite. The “Varroa App”,^1^ for example, is designed to help beekeepers with treatment, or the “Varroawetter” which provides information on optimal weather conditions for varroa treatment for all regions in Germany.^2^ Although these are valuable tools, there is often a lack of data that would allow a more accurate and realistic picture of the treatment situation. The public sector is currently unable to provide more data, especially since trials would be necessary, which would require a large number of colonies and replicates that would have to be maintained for many years.

Mechanistic modelling provides a cost‐effective alternative to empirical studies, where a larger number of scenarios can be tested in silico that would be prohibitively expensive otherwise (Becher et al., 2013; Henry et al., 2017). Among a large number of published honeybee models, the BEEHAVE model (Becher et al., 2014) appears to be the most sophisticated. It includes a detailed foraging module, the option to load a realistic landscape and a varroa and virus model that enables various beekeeping practices. BEEHAVE was positively evaluated by the European Food Safety Agency (EFSA PPR Panel, 2015) and has been used to answer a variety of questions: Consequences of pesticide impacts at the colony level, both in hypothetical (Reiner et al., 2022; Rumkee et al., 2015; Thorbek et al., 2017a,b) and real‐world scenarios (Schmolke et al., 2019, 2020); and numerous others (Abi‐Akar et al., 2020; Agatz et al., 2019; Bulson et al., 2021; EFSA, 2021; Henry et al., 2017; Horn et al., 2016, 2021; Requier et al., 2019). Although the original BEEHAVE version (Becher et al., 2014) includes varroa treatment and a later version (BEEHAVE for BeeMapp, 2016) includes repeated treatments, drone brood removal, and mite reinvasion (referred to as reinfestation in BEEHAVE), not all varroa treatment options can be simulated.

To evaluate the efficacy of different varroa control measures including organic acids and drone brood removal, we extended the existing honey bee model BEEHAVE (Becher et al., 2014, 2016 version). A new module was implemented that better reflects treatment practice in Germany, i.e., allowing multiple applications of drone brood removal and organic acid applications. In the following, we briefly describe BEEHAVE and then the new module in detail. We subsequently present the varroa dynamics simulated by the model and link our results to empirical findings from the literature. Particular attention is paid to the examination of parameters that determine or limit the effectiveness of the varroa treatment carried out, such as proportion of drone pupae removed, mite preference for the invasion of drone and worker brood cells for reproduction, reinvasion of mites, or the number of acid treatments. This is because in the model based on expert assumptions and literature data, the effectiveness of drone brood removal was significantly higher than indicated for real bee colonies. Finally, we discuss possible reasons for this discrepancy, suggesting that certain details of the interactions between varroa and honey bees are not yet well understood or are inadequately represented in the model.

## 2 METHODS

The original BEEHAVE model and a user's guide and manual are publicly available at https://www.beehave‐model.net. The model is programmed and executed using the freely available software platform NetLogo (Wilensky, 1999). For this study, the BEEHAVE implementation BEEHAVE_BeeMapp2016 (available at www.beehave‐model.net) was updated to be used in NetLogo 6.2 (Wilensky, 1999). The resulting program and a full model description following the standard format ODD (Grimm et al., 2006, 2020) are included in the COMSES model library (Schödl et al., 2022).

### 2.1 The model BEEHAVE

BEEHAVE was developed to examine how different stressors, alone or in combination, impact the vitality and survival of a single honey bee colony (Becher et al., 2014). Stressors may include varroa mites and virus infections, impaired foraging, pesticides, or suboptimal beekeeping practices. BEEHAVE consists of three main components. The colony model is cohort‐based and comprises the daily changes of the bee colony structure, i.e. the brood, workers, and drones. These dynamics are driven by the daily egg‐laying rate of the queen bee.

The foraging model is individual‐based (here, one individual in the model represents a so‐called superindividual, i.e. 100 bees) and simulates the foraging behaviour of the bees, including scouting for new rewarding floral resources in the landscape and recruitment of foragers via waggle dance. Foragers collect nectar and pollen in the given landscape, but only when weather conditions are favourable. The landscape is represented as a list of fields or patches, which sooner or later in the year provide nectar and/or pollen. Each patch is characterised by distance from the beehive, probability of detection by foragers, flowering period, and nectar and pollen provisioning.

The mite model manages the mite population in the beehive and is based on the established mite model by Martin (1998, 2001). Mites are described as individuals and can either be inside the brood cells or phoretic, i.e. attached to an adult bee. In the model, mites themselves do not affect the bees, but they transmit viruses, which then increase the mortality of the infected pupa or adult bee. The reproduction of the mites takes place in the brood cells. The default option in BEEHAVE allows a maximum of four mites per brood cell, regardless of whether it is a worker or drone brood cell.

### 2.2 The new varroa control module

In the following, we describe additions to the BEEHAVE model that were necessary to better describe varroa control measures. Since drone brood removal is an important measure, we had to update the previous modelling of drone egg production. In BEEHAVE, the daily number of eggs laid by the queen is based on assumptions made in the honey bee population model HoPoMo (Schmickl & Crailsheim, 2007). Eggs are laid continuously throughout the brood season of honey bees. These laid eggs are mostly fertilized, i.e. they develop into workers. Drones are only present in the bee colony during the summer months. In the original BEEHAVE version, 4% of the number of worker eggs laid during summer are drone eggs.

**FIGURE 1:**
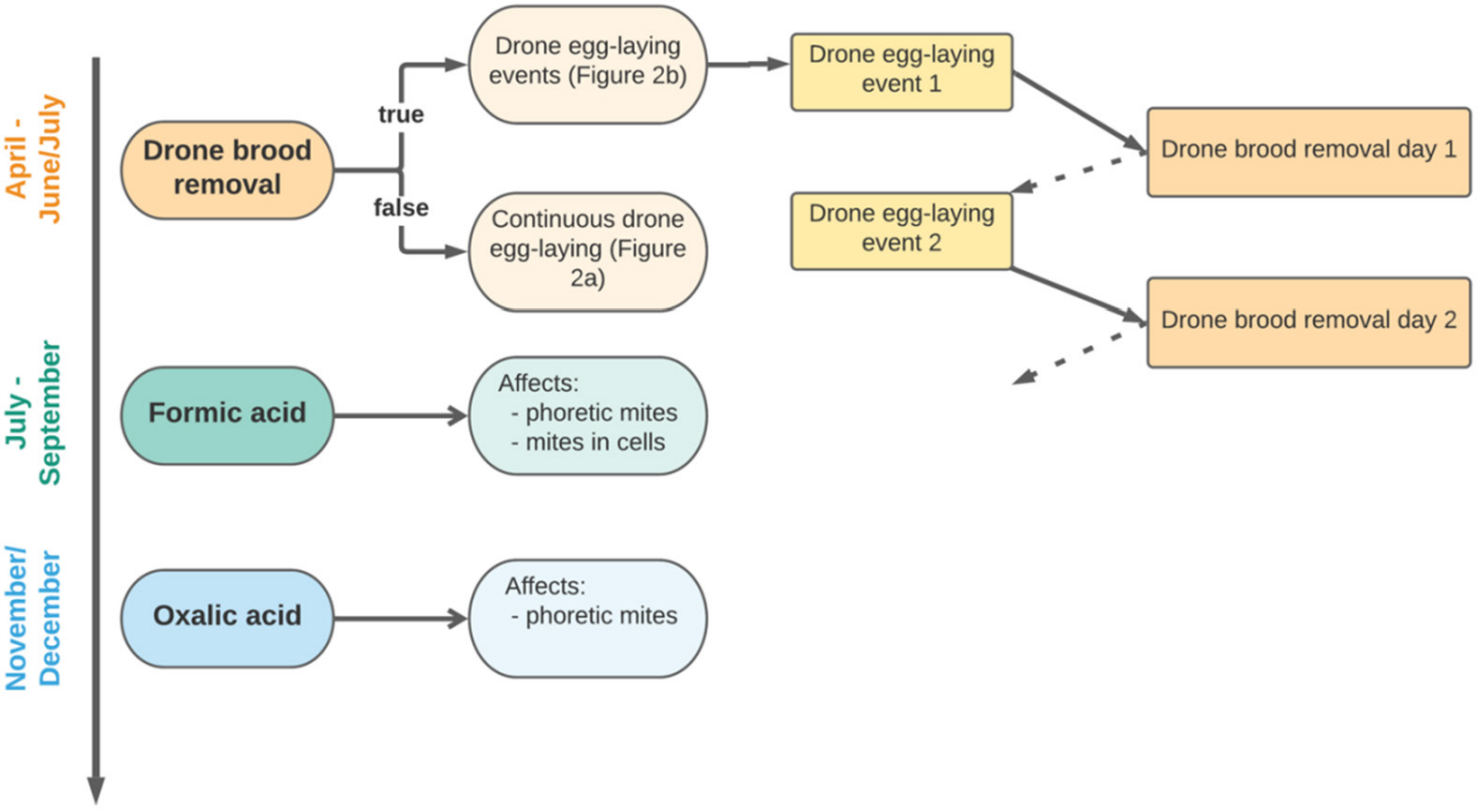
Overview of the underlying process for the implementation of drone brood removal, its connection to egg‐laying, and acid treatments in BEEHAVE.

We adapted the egg‐laying process in BEEHAVE to represent the pulsed production of drone eggs. If the drone egg‐laying season has started, the queen will lay eggs in the drone brood cells on the specific frame. After the drone brood cells are capped, the frame is removed by the beekeeper and in the model the corresponding drone brood is removed. The dependency of the drone egg‐laying events with the drone brood removal is illustrated in Figure 1. The number of drone egg‐laying events and drone brood removal days depends on how often the removal should take place during a year in BEEHAVE. Note that due to the way drone brood removal is implemented in BEEHAVE, during a drone brood removal event only capped drone brood (i.e. drone pupae) is removed.

We assumed that approximately 3000 brood cells fit on one drone brood frame. If drone brood removal is applied, the queen lays eggs on the imaginary drone brood frame with the start of the drone egg‐laying season. For a maximum of five days, the queen lays predominantly drone eggs. A background worker egg‐laying of 10% of the total number of eggs on each of these days and the upper limit of 5 days was introduced to avoid a significant drop in the number of worker bees over the season. The actual number of drone eggs laid on a drone frame could be below 3000, depending on the queen's current overall egg‐laying rate. During the period of drone breeding, i.e. calendar day 105 to 182, the queen still lays 5% of her eggs as drone eggs on the days the drone frame was not available. This reflects that even if a colony is managed with a drone brood frame, some drone brood cells are still built outside of that frame. As a consequence, about 10% of all eggs in a simulation year are drone eggs. The resulting number of adult drones was then compared to empirical data by Dettli (2019), which match in number (figure not shown). It should be noted that the proportion of drone to total eggs in a simulation year is higher than in the original BEEHAVE (version 2014) with <4% drone eggs.

If the drone brood is not removed, egg laying will function as in the original BEEHAVE version. The continuous drone egg‐laying simulates a bee colony that is not provided with a trap frame by the beekeeper. Such a colony should have at least as much drone brood as a colony managed using drone brood frames (Büchler, 1996; Dettli, 2009; Liebig, 1997). To achieve the proportion of 10% drone eggs per year, for continuous drone egg‐laying within the drone egg‐laying season 20% of daily eggs laid are drone eggs in the model. The off‐season is 100% worker eggs.

Treatments with formic and oxalic acid are defined by the start of the application and its duration (in days). Both acids are only effective for a few days in the bee colony. In contrast to the use of synthetic acaricides, they have a short but strong effect (Liebig et al., 2004; Rosenkranz et al., 2010). In our BEEHAVE version, two treatment periods can be specified for formic acid (FA), whereas for oxalic acid it is just one. Formic acid affects mites in brood cells and phoretic mites with different efficacies. The latter is comparatively higher (Calderón et al., 2000; Steube et al., 2021). Oxalic acid only kills phoretic mites and therefore only a phoretic efficacy is specified.

### 2.3 Initialisation, simulation experiments, and output analysis

To study the effects of varroa mites and their treatment on a bee colony without an additional stressor, the nectar and pollen supply for the bee colony was characterised by a highly stylized landscape with two food patches. This landscape is the default setting, as defined in the original BEEHAVE publication (Becher et al., 2014), and results in sufficient food supply over the entire foraging season. To avoid interactions of mite dynamics with foraging conditions, the weather scenario was set to a constant eight‐hour foraging period per day. As in Horn et al. (2016), the foraging season was limited to calendar days 80 to 290 (corresponding to 21 March to 17 October). Drones only occur during the summer months and therefore the drone egg‐laying season was altered, so that drone eggs are present from 15 April (calendar day 105) to 1 July (calendar day 182). Other drone stages exist in the colony until their natural death in the model.

The default mite reproduction model in BEEHAVE was used with a maximum of four mites per brood cell (according to Martin, 1998). Varroa mites enter drone cells with a higher probability, which is the main reason for removing drone brood and utilising it as mite trap. The standard value used for the preference ratio *pr* of mites regarding the invasion of drone and worker brood cells for reproduction is 11.6, which means that mites are 11.6 times more likely to enter drone brood cells than worker brood cells. The preference ratio *pr* of 11.6 is calculated from Equation 1 with factorDrones=6.49 and factorWorkers=0.56 (after Boot et al., 1995):

(1) $$\mathrm{pr}=\frac{\text{factorDrones}}{\text{factorWorkers}}$$

Since the exact value of the preference ratio *pr* is subject to large uncertainty, we changed *pr* during the analysis by leaving the factorWorkers at the default value and varying the factorDrones. In a bee colony managed with a drone brood frame, drone brood does not only occur on this particular frame, but can be found anywhere else in the beehive. To account for this issue, a parameter was introduced into BEEHAVE to be able to scale the proportion of drone brood removed on a drone brood removal day. According to expert opinion, about 80% of drone brood in a bee colony on the final day of egg laying on the drone brood frame are on this frame. The other 20% are scattered over the remaining frames (unpublished data from V. Mustafi and C. Otten, and expert knowledge from S. Berg).

Default initial settings of BEEHAVE were utilised otherwise (Table 1), i.e. the simulations started with 10,000 worker bees on calendar day 1. To allow the model to settle, a burn‐in phase of one year was used before introducing mites into the bee colony. One year was considered sufficient due to favourable foraging and weather conditions in the simulations. On the first day of the second simulation year (day 366) 40 healthy mites and 10 mites capable to transmit DWV were introduced. Consequently, varroa treatments started in the second year. Since BEEHAVE is a stochastic model, i.e. it includes random distributions and probabilities, simulations with the same setting were run 10 times.

**TABLE 1:** BEEHAVE parameter; includes the parameters that differ from the standard BEEHAVE values (bold) and the added parameters for the new implementations

Parameter	Value	Unit	Description
Weather	**Constant**	/	
SEASON_START	**80**	[d]	Start of foraging season (corresponds to 21 March)
SEASON_STOP	**290**	[d]	Stop of foraging season (17 October)
DRONE_EGGLAYING_START	**105**	[d]	Start of drone egg laying season (15 April)
DRONE_EGGLAYING_STOP	**182**	[d]	Stop of drone egg laying season (1 July)
N_INITIAL_MITES_HEALTHY	**40**	# mites	Added after one‐year burn‐in phase
N_INITIAL_MITES_INFECTED	**10**	# mites	Added after one‐year burn‐in phase
Virus	DWV	/	Deformed Wing Virus
MiteReproductionModel	Martin	/	According to Martin (1998)
useDBRdaysAsDroneEgglayingTime	true	boolean	Automatically sets the next start day of a drone egg‐laying event one day after a drone brood removal day; e.g. drone brood removal day 119, start of next drone egg‐laying event is day 120; corresponds to reinserting the drone brood frame after its removal by the beekeeper
cellsDroneBroodFrame	3000	#	Assumed mean number of drone brood cells on a drone brood frame (both sides)
maxDroneFrameEgglayingDuration	5	[d]	Maximum days of one drone egg laying event to avoid a significant drop in colony, i.e., worker, strength
workerEggsProportionOnDroneEgglayingDays	0.1	Proportion	Background worker egg laying to avoid a siginficant drop in worker strength. This was achieved with setting the background worker egg laying rate” to 10%
droneEggsProportionNoDroneEgglayingDay	0.05	Proportion	Background drone egg proportion of 5%
droneEggsProportionNoDBR	0.2	Proportion	In drone egg laying season: proportion of 20% drone eggs per day of total egg number on this day
propRemoveDronePupae	Default: 0.8		Defines what proportion of drone pupae is removed on each drone brood removal day; does not correspond to all drone pupae, but the drone pupae with ages found on the drone brood frame
formicAcidVarroaTreatment	True, if used	Boolean	
formicAcidTreatmentDay1‐1	211	[d]	Corresponds to July 30; start of application is then July 31
formicAcidTreatmentDuration1	2	[d]	
formicAcidEfficiencyPhoretic1	0.4	/	Proportion to reduce phoretic mite numbers
formicAcidEfficiencyBroodcells1	0.2	/	Proportion to reduce mite numbers in brood cells
formicAcidTreatmentDay2‐1	242	[d]	Corresponds to August 30; start of application is then August 31
formicAcidTreatmentDuration2	3	[d]	
formicAcidEfficiencyPhoretic2	0.3	/	Proportion to reduce phoretic mite numbers
formicAcidEfficiencyBroodcells2	0.1	/	Proportion to reduce mite numbers in brood cells
oxalicAcidVarroaTreatment	False	Boolean	
oxalicAcidTreatmentDay‐1	339	[d]	Not used in this study; default value is set to day 339
oxalicAcidTreatmentDuration	−10		Not used, therefore −10 as default value
oxalicAcidEfficiency	0		

Our study focused on the representation of the drone brood removal in BEEHAVE. Therefore, we ran two removal scenarios with (i) 2‐week‐gap between removals and (ii) 3‐week‐gap between removals, as well as a reference scenario with no removals. Mite pressure *mp*(*d*), i.e. the number of mites on a given day *d*, was used as a metric to quantify the efficacy of drone brood removal. The reduction in mite pressure between a removal scenario and the reference scenario was calculated as:

(2) $$\mathrm{Reduction}\;\mathrm{mp}\left(d\right)=\frac{\left(\#\mathrm{mites}\;\mathrm{in}\;\mathrm{reference}\;\mathrm{scenario}\left(d\right)-\#\mathrm{mites}\;\mathrm{in}\;\mathrm{DBR}\;\mathrm{scenario}\left(d\right)\right)}{\#\mathrm{mites}\;\mathrm{in}\;\mathrm{reference}\;\mathrm{scenario}\left(d\right)}$$

where #mites are the mean over the 10 runs and *d* is the calendar day.

The parameters for the acidic treatments were systematically varied to determine a parameter set so that on calendar day 365 in the second year of the simulation there are a maximum of 50 mites in the bee colony. This number of mites for the overwintering period is a recommended target of the described varroa control measures (Genersch et al., 2010; Liebig, 2001). However, it should be noted that this threshold is dependent on the size of the bee colony. Two assumptions, based on expert assessments, constrained the parameter variation: Firstly, formic acid has a stronger effect on phoretic mites than on mites in brood cells (Calderón et al., 2000; Steube et al., 2021). Since the formic acid treatment is never 100% effective, a second treatment is scheduled four weeks after the first one to compensate for this shortfall (Pietropaoli & Formato, 2017; Steube et al., 2021). Due to the advanced temperature drop in late summer, the FA dispenser should remain in the colony for a longer period than during the first treatment. Hence, the application volume of formic acid is higher, allowing for a broader treatment spectrum to ensure sufficient evaporation performance.

### 2.4 Empirical patterns

Here we briefly describe the empirical patterns that we wanted to reproduce with the model. The varroa control measures implemented should be evaluated based on their ability to reproduce the following two criteria: (1) Mite pressure reduction of 50–67% through drone brood removal (expert opinion). Calis, Boot, and Beetsma (1999), Calis, Fries, and Ryrie (1999) and Charrière et al. (2003) indicate a reduction in mite pressure of up to 50%. It should be noted that drone brood removal alone is not sufficient to control varroa (e.g. Charrière et al., 2003). (2) When comparing a two‐ and a three‐week interval between the drone brood removal days, there should be no difference in the mites collected, as no drone pupae hatched on the frame before 21 days either. This criterion served as a consistency test.

Moreover, since BEEHAVE is considered realistic enough to represent key features of real honey bees, we expected that it would be possible to find parameter combinations for drone brood removal and acid treatment that allow for a successful control, i.e. the colony does not have more mites at the end of the second year than at the beginning of that year.

### 2.5 Statistical analysis

Analysis, handling and visualisation of the simulation output was carried out with *R* 4.0.3 (R Core Team, 2020); for details see result part and the scripts that can be downloaded from COMSES model library (Schödl et al., 2022). For the analysis *R* packages “here” (Müller, 2020), “plyr” (Wickham, 2020), “dplyr” (Wickham, Chang, et al., 2021), “ggpubr” (Kassambara, 2020), “pacman” (Rinker & Kurkiewicz, 2019), “knitr” (Xie, 2021), “conflicted” (Wickham, 2019), “Cairo” (Urbanek & Horner, 2020) were used. Plots were generated using the package “ggplot2” (Wickham, François, et al., 2021).

The Wilcoxon‐Mann–Whitney test was used as a non‐parametric statistical test to check whether two modes of drone brood removal are different (see Figure 5 for details). A significance level of α=0.05 was used. A *p*‐value >.05 is not significant and denoted with the letters ‘ns’.

## 3 RESULTS

### 3.1 Dynamics without drone brood removal

**FIGURE 2:**
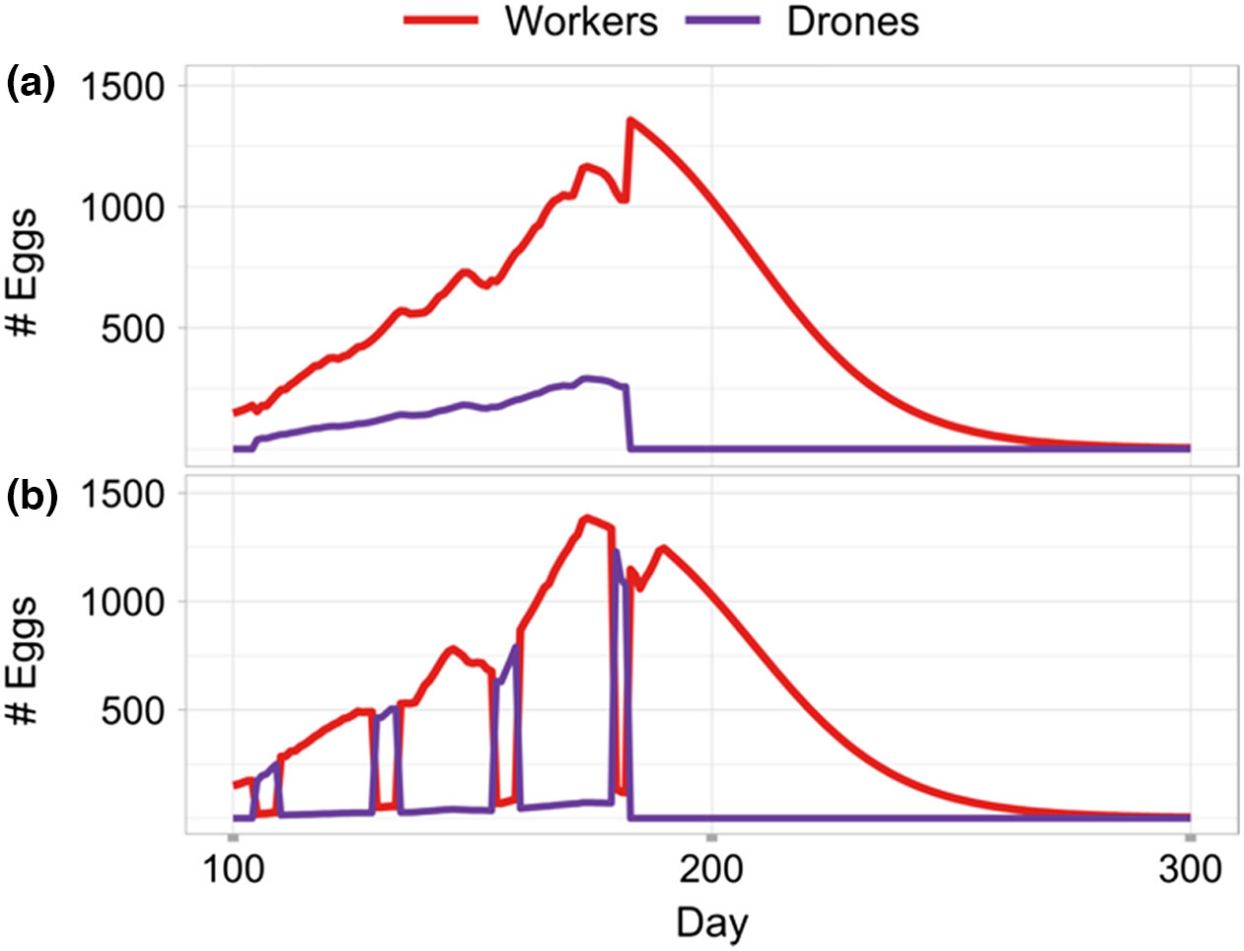
Exemplary time series of eggs laid per day over one year in BEEHAVE; (a) the original continuous egg‐laying of drone and worker eggs, (b) modelling of drone egg‐laying in drone egg‐laying events: during these days most of the eggs laid are drone eggs, on the other days the majority of the eggs are worker eggs (maximum number of eggs per day is based on the model HoPoMo; Schmickl & Crailsheim, 2007).

In BEEHAVE, the dynamics of the bee colony is driven by the queen's egg‐laying. The new implementation of drone brood removal resulted in two possible ways of drone egg‐laying: either continuous, as in the original BEEHAVE model, or organised in drone egg‐laying events (Figure 2).

**FIGURE 3:**
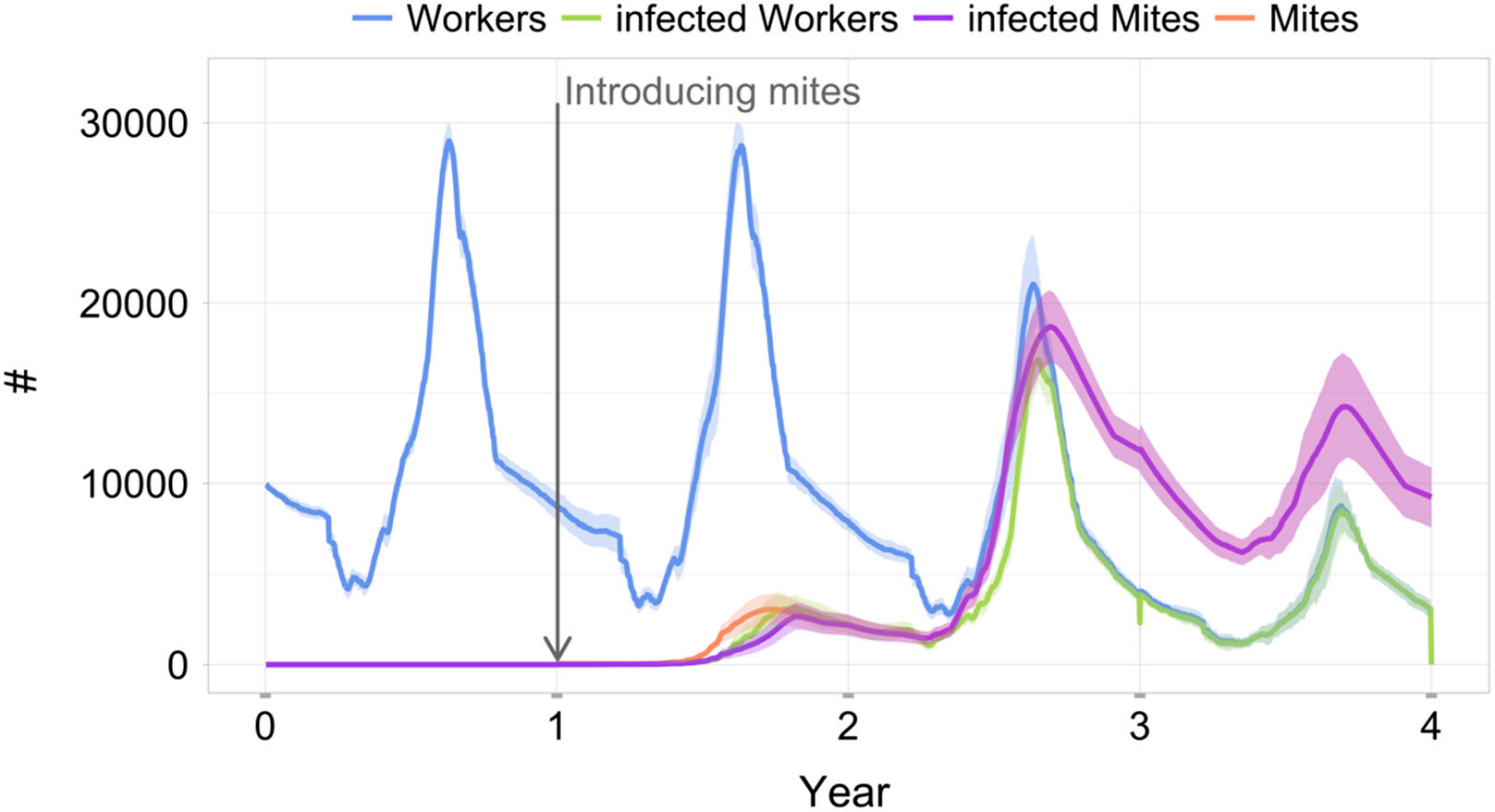
Dynamics of bee colonies infested with varroa mites capable to transmit DWV (Deformed Wing Virus) (*n* = 10, i.e., 10 simulations with one bee colony each, mean ± SD). Four colonies collapsed after two years after the introduction of mites (40 healthy and 10 infectious on day 366), and the remaining six colonies after three years after the mite introduction. Therefore, in the figure, after year 3, the mean and SD only comprise the remaining six colonies. On the y‐axis the number of adult bees and mites are presented. BEEHAVE simulation result in very high mite numbers (>10,000 individuals).

For a healthy bee colony, i.e. without mites and viruses, the colony size (worker bees) in the model simulations showed a typical pattern with a peak in summer and a linear decline in winter. These patterns are largely driven by the queen's egg‐laying rate and the mortality of winter bees (first year in Figure 3). This dynamic changed after the introduction of mites into the bee colony at the start of the second year, of which 40 mites were healthy and 10 capable to transmit DWV. More and more mites, as well as workers, became infected until all of them carried the virus. Two to three years after the introduction of mites, bee colonies collapsed due to winter mortality. In BEEHAVE, winter mortality is defined by having a total number of worker bees below the threshold of 4000 bees at the end of a simulation year (calendar day 365) (Martin, 2001; from Free & Spencer‐Booth, 1958 and Harbo, 1983).

### 3.2 Drone brood removal

To test whether the effects of simulated drone brood removal match the empirical effects, a two‐week gap scenario and a three‐week gap scenario were compared (Table 2, scenario 2‐ and 3‐week‐gap). For both scenarios, the starting day for calculating the gap between removal days was the start of the drone egg‐laying season on calendar day 105. Drone brood removal was performed four times a year.

**TABLE 2:** Drone brood removal scenarios in BEEHAVE; parameter DroneBroodRemoval in BEEHAVE is set to true. droneEgglayingTime and Removal day are provided in Julian calendar days.

Event scenario	Parameter	1	2	3	4
2‐week‐gap					
	droneEgglayingTime	105	120	135	150
	Removal day	119	134	149	164
3‐week‐gap					
	droneEgglayingTime	105	127	149	171
	Removal day	126	148	170	192

**FIGURE 4:**
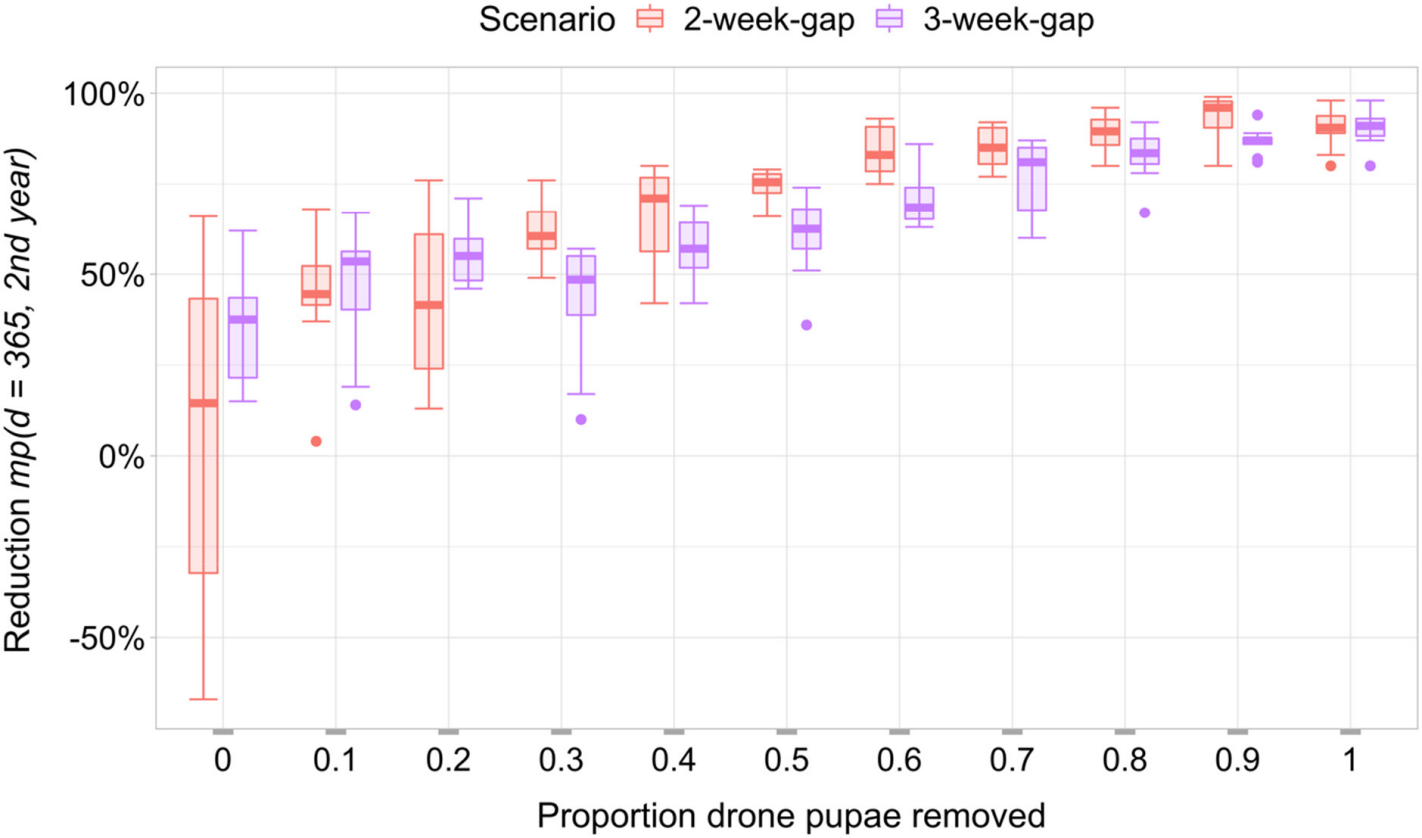
Mite pressure reduction and varying the proportion of drone pupae removed on a drone brood removal day for bee colonies infested with varroa mites when drone brood removal was applied (scenario 2‐week‐gap and 3‐week‐gap) in comparison to bee colonies without treatment (comparison day: day 365, 2nd year); for reduction *mp*(*d*) see Equation 2.

To better understand the influence of the parameter for the proportion of drone pupae removed during a removal event, this parameter was varied to a large extent (Table 2, Figure 4). Our known estimate for this parameter is 80%, and since modifications to this parameter did not change the overall reduction in mite pressure (Figure 4), we used 80% for all the other simulations.

**FIGURE 5:**
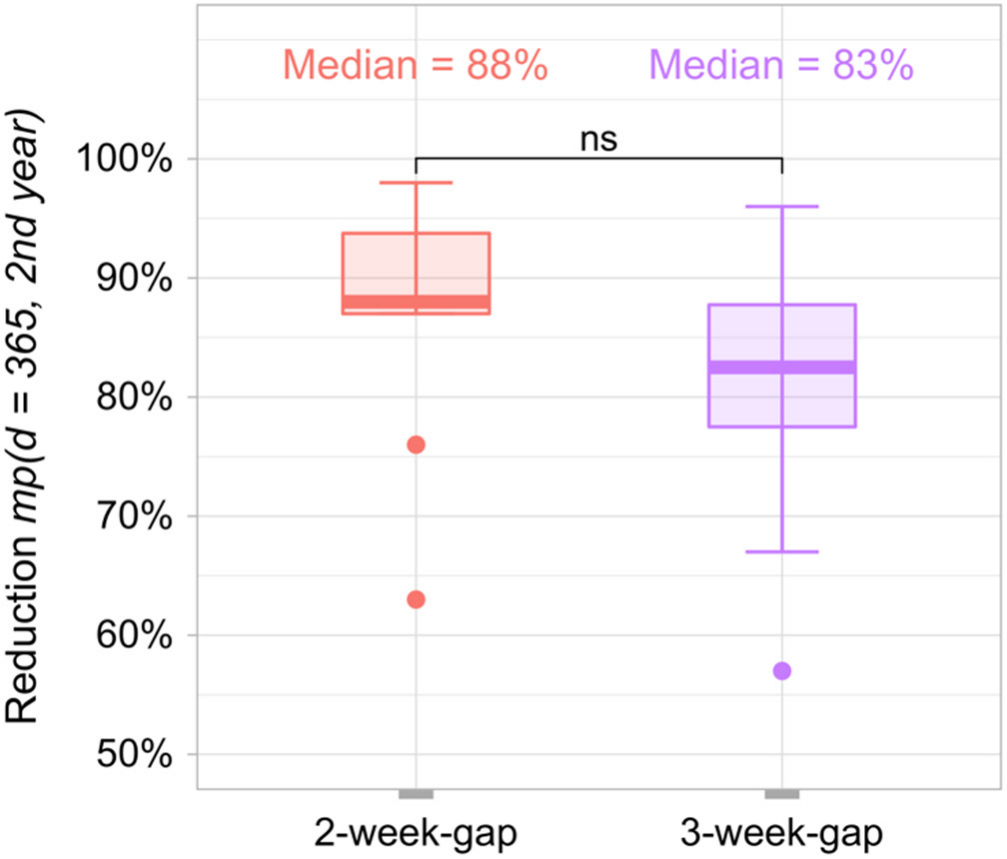
Mite pressure reduction for bee colonies infested with varroa mites when drone brood removal was applied (scenario 2‐week‐gap and 3‐week‐gap) in comparison to bee colonies without treatment (comparison day: day 365, 2nd year); for reduction *mp*(*d*) see Equation 2; statistical significance: Wilcoxon‐Mann–Whitney test, ns = *p*‐value not significant; boxplot each comprises *n* = 10 simulations, i.e. 10 bee colonies. Note that new simulations were carried out in comparison to Figure 4.

The mite pressure reduction for the 2‐week‐gap and 3‐week‐gap drone brood removal scenarios for a proportion of drone pupae removed of 80% is in more detail in Figure 5. Mite pressure reduction is similar for both scenarios, with a median of 88% for the 2‐week‐gap and 83% for the 3‐week‐gap scenario. It is worth noting, that according to bee expert opinion, consistent drone brood removal reduces mite pressure by 50% (Criterion 1, Section 2.4).

Considering criterion 2, the reduction in mite pressure is not visually distinct and not statistically significant among the scenarios, as the values are equal ranges and far from the target value (Wilcoxon‐Mann–Whitney test, *p*‐value <.05; significance was not artificially forced by unrealistic large numbers of replicate simulations; White et al., 2013). Thus, our simulation results are consistent with expectations from the mite ecology that there should be no detectable difference between 2‐week‐gap and 3‐week‐gap treatment.

### 3.3 Drone brood removal: adjustments

Since drone brood removal, as implemented in our module, was more effective than generally assumed, two possible adjustments were identified in BEEHAVE to dampen the efficacy of this measure. To analyse these adjustments only the 2‐week‐gap scenario was considered, as this is the standard gap between removal days according to bee experts.

**FIGURE 6:**
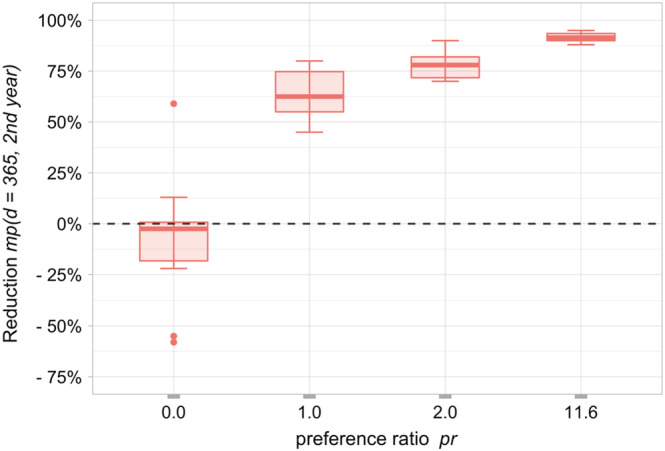
Reduction in mite pressure due to the variation of the mite preference for the invasion of drone and worker brood cells for reproduction via the preference ratio *pr* (Equation 3), where factorWorkers was left at the default value 0.56 and factorDrones was varied; means (*n* = 10) drone brood removal scenario 2‐week‐gap and reference scenarios without drone brood removal.

The first adjustment was the preference ratio *pr* of mites regarding the invasion of drone and worker brood cells for reproduction. This ratio was varied to quantify its influence on the reduction of the mite pressure *mp* (Figure 6). With a preference ratio *pr* of 0, mites do not enter drone brood cells and are expected to result in a 0% reduction of mite pressure *mp* accordingly. The deviation from 0% in the boxplot is due to the comparison of pulsed and continuous drone egg‐laying with and without drone brood removal. Mite pressure reduction was still higher than the reduction expected by experts for a preference ratio of 1.

**FIGURE 7:**
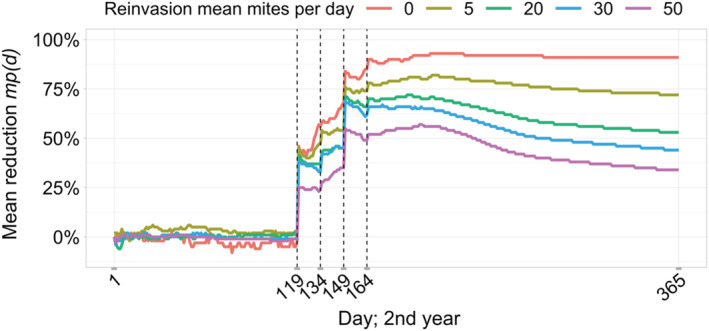
Mite reinvasion was added in BEEHAVE to reduce the efficacy of drone brood removal and its effect on the mite pressure reduction is shown; mites and reinvasion were introduced in the second year of the simulation. Reinvasion is modeled with a Poisson distribution with mean mites (legend) entering the bee colony with returning bees per day; drone brood removal scenario: 2‐week‐gap (*n* = 10, i.e., 10 bee colonies for each reinvasion value).

The second adjustment for the efficacy of drone brood removal was the reinvasion of varroa mites by forager bees returning to their colony carrying mites with them. In BEEHAVE, the number of mites that are introduced into the bee colony per day is determined via a Poisson distribution with a specification of the mean value. The reinvasion method implemented in the 2016 BEEHAVE version has not yet been applied in a published study. Increasing the mean number of mites per day re‐entering the bee colony steadily reduces the efficacy of the drone brood removal (Figure 7). However, mite reductions of around 50% are only reached, when 20 to 30 new mites per day enter the colony.

### 3.4 Good beekeeping practice

**FIGURE 8:**
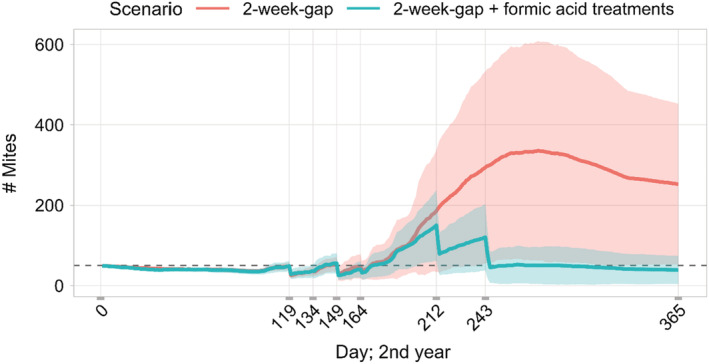
Mite numbers over the first year of varroa infestation comparing varroa control with just drone brood removal (treatment days 119, 134, 149, and 164) and adding formic acid treatment (treatment start days 212 and 243) (*n* = 10, i.e., 10 bee colonies for each scenario, mean ± SD); dashed line at # Mites = 50. For specifications for formic acid treatment parameters see Table 1.

Although we showed that the drone brood removal is highly effective in terms of reducing mite pressure, mite numbers in the bee colony are still too high at the end of the year in which only drone brood removal was used (Figure 8, red line). We set a target of at most 50 mites in the colony after all varroa control measures. Comparing the number of mites between treated and untreated colonies emphasises again the high efficacy of the drone brood removal: without treatment, the maximum number of mites is above 3000 in the year after the introduction of mites (Figure 3, orange line, second year).

Accordingly, starting from the drone brood removal scenario with a two‐week gap between removal days, the organic acid treatments were added one after the other, so that at the end of the second year the number of mites is below 50 (regardless of their infection status). This can be obtained by adding two formic acid treatments and no oxalic acid treatment to the drone brood removal measure (Figure 8, blue line). Initial percentages for the formic acid treatment, by which mite numbers were reduced on each treatment day are displayed in Table 1.

**FIGURE 9:**
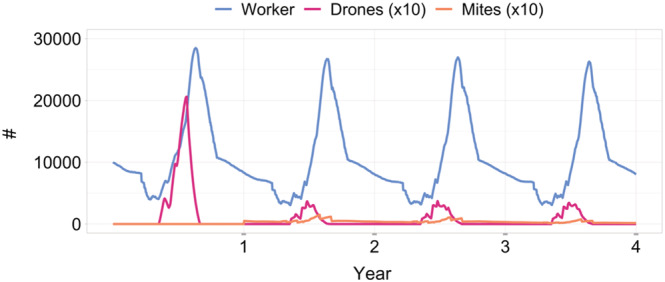
Bee colonies infested with varroa mites capable of transmitting DWV, and treated according to Good Beekeeping Practice with drone brood removal and formic acid treatments; drone and mite numbers are increased by a factor of 10 for clarity (mean of *n* = 10 simulations, i.e., 10 bee colonies); mites and varroa control strategy as Good Beekeeping Practice were introduced at the start of the second year after a one‐year burn‐in phase.

With this varroa control strategy, which is applied every year in the same way, the bee colonies are doing well with an average of about 27,500 bees as the peak colony strength every year in a longer simulation run (Figure 9, blue line). The number of mites is kept consistently low over the years (Figure 9, orange line). Comparing the number of drones in year one to the subsequent years, the effect on drone numbers of the drone brood removal is visible (Figure 9, pink line).

In summary, drone brood removal as currently simulated requires only two treatments with formic acid to keep mite pressure low. Under the optimal conditions of DBR implemented in our model, a colony can overwinter successfully. Please note that winter treatment with oxalic acid, which is also part of Good Beekeeping Practice, is not yet applied in the simulations at this time.

## 4 DISCUSSION

Based on the existing BEEHAVE model, varroa control strategies of the German federal states according to Good Beekeeping Practice were newly implemented. We focused our study on the integration and analysis of drone brood removal with a drone brood frame. In combination with organic acid treatments, drone brood removal forms a holistic varroa control strategy without residues of synthetic pesticides in hive products. This strategy was applied to mite‐infested colonies in BEEHAVE.

In reality, drone brood removal usually reduces mite pressure in a colony up to 50% until mid‐season compared to a colony without this intervention (Calis, Boot, & Beetsma, 1999; Calis, Fries, & Ryrie, 1999; Charrière et al., 2003; Wantuch & Tarpy, 2009; Whitehead, 2016). In BEEHAVE, our 2‐week gap and 3‐week gap scenarios achieved about an 85% reduction in mite pressure in the median at the end of the year after mites were introduced into the colony (Figure 5).

Explaining this discrepancy between reported and simulated efficacy is difficult given the wide range of drone brood removal efficacy in empirical studies. Schulz et al. (1983) found different dynamics depending on the initial level of mite pressure in a bee colony. When they removed drone brood in colonies that started with high varroa numbers, a reduction in mite pressure of only 15% occurred. However, in colonies with low varroa numbers, drone brood removal without further varroa treatment kept the mite pressure low. Since, we started our experiment with a fairly low pressure of 50 mites, the result of the model seems reasonable. In addition, Schulz et al. (1983) found that the group of colonies managed with drone brood removal for two years had an average mite pressure of 266 mites per colony in the fall after this time. According to the authors, these colonies would have survived the next year without further treatment. There appear to be empirical cases where drone brood removal is a sufficient varroa treatment in some years.

In contrast, Rademacher (1990) reported a very low drone brood removal efficacy of 10%. whereas much higher reductions in mite pressure were documented by Charrière et al. (1998, 2003) and Radtke and Neuberger (2008). Experiments in Switzerland demonstrated an efficacy of 47–73%, with the higher values obtained in colonies from which little honey was extracted (Charrière et al., 1998, 2003). So far, only the efficacy reported by Radtke and Neuberger (2008) is comparable to the reduction in mite pressure achieved in BEEHAVE, at 85%. According to the authors, this value can be accomplished under good forage conditions, which we implemented in BEEHAVE for this study.

Rosenkranz and Engels (1985) observed that colonies in which drone brood was removed did not show a critical threshold of mites in the colony until one to two years later, compared to control colonies. This threshold ranges from an infestation rate of 7% for winter bees (Liebig, 2001) and about 30% of adult bees in summer (Fries et al., 2003; Rosenkranz et al., 2006). Overall, drone brood removal was insufficient as a sole varroa control over several years (Rosenkranz & Engels, 1985), consistent with our simulations. It only delayed the collapse of colonies if no further measures were taken, confirming the results of Wantuch and Tarpy (2009). At this point, it must be mentioned that drone brood removal requires specific and detailed implementation to be effective in practice. Starting the removal of frames (with late larval stages or fully capped) at the beginning of the season at well‐defined intervals (2–3 weeks) will ensure the effectiveness of the method. This allows between 3 and 5 frames to be removed (Odemer et al., 2022; Whitehead, 2016).

Calis, Boot, and Beetsma (1999), Calis, Fries, and Ryrie (1999) developed a varroa mite population model, in which they investigated the effect of the removal of drone brood on the number of mites in a bee colony. It was assumed that 1500 eggs are laid on a single drone brood frame. This corresponds to half of the number of brood cells per drone brood frame used in this study. When drone brood was removed twice in the model (each year on June 1 and July 1), a reduction of 89% in the maximum mite count was observed in the third year. This agrees very well with the 90% reductions we simulated. However, the authors chose different days (i.e., June 1 and July 1) to compare mite counts. That two independent models yield very similar predictions that likely overestimate the effectiveness of drone brood removal may indicate that we are lacking important understanding of the role of drone brood removal.

To harmonize the model results with empirical observations and to achieve more biologically relevant simulations, the mites' preference ratio regarding the infestation of drone and worker brood cells for reproduction was examined. In BEEHAVE, the value by Boot et al. (1995) is used as default: mites are 11.6 times more likely to invade drone than worker brood cells. However, a wide range of this ratio appears in the literature. While Fuchs (1990) reported values ranging from 0.94 to 30.6, Calis et al. (1993) found a ratio from 7.7 to 15.3. On average, mites prefer drone cells over worker cells by a factor of 8.5 (Schulz (1984): 8.6 and Fuchs (1990): 8.3). This preference changes with the season and depends, among other things, on the ratio of worker and drone brood cells present in the hive. We choose the default value of BEEHAVE, 11.6, but varied it over a wide range. The model seems to be rather insensitive to this parameter variation (Figure 6).

Reinvasion of varroa mites into a bee colony by mite‐infested foragers was examined as another adjustment possibility of the model (Kulhanek et al., 2021; Sakofski et al., 1990; Sakofski & Koeniger, 1988). Mean values of daily mites introduced into the colony ranging from 0 to 50 were investigated (Figure 7). To achieve the targeted 50% mite pressure reduction (according to expert opinion), an average of 20 to 30 mites per day had to enter the beehive in the simulation.

In fact, mite reinvasion numbers follow a seasonal pattern with low values in spring, increasing values in summer and autumn, and decreasing values towards winter. Greatti et al. (1992) found seasonal entries between 1.6 and13.7 mites/day/colony from June to early August. In September and October, the invasion values jumped sharply with an average of up to 75.6 mites/day/colony. Frey and Rosenkranz (2014) studied the reinvasion of varroa mites at two sites, one with low bee density, and the other with high bee density. At the latter site, the highest weekly mean value of mite invasion in previously treated and mite‐free colonies was about 65 mites (time: end of August). The reinvasion at the site with low bee density remained below 20 mites. In contrast, other studies have found almost no reinvasion of mites from surrounding colonies (Goodwin et al., 2006; Neumann et al., 2000). Hence, values of 20–30 mites on average per day that were implemented in the model are not unrealistically high. The reinvasion in BEEHAVE should nevertheless be adjusted to the seasonal pattern found in the empirical data for further use.

Good Beekeeping Practice including biotechnical mite control is widespread in Germany, but treatment success varies in some cases (Jacques et al., 2017). The interactions between treatment parameters such as timing and frequency of drone brood removal, formic acid treatment, and oxalic acid treatment with environmental factors and colony condition are not always easy to overview and are very complex to implement in the BEEHAVE model. Thus, more treatment‐specific data are needed to form a realistic representation of Good Beekeeping Practice and other aspects of different operating modes. Since there is a great variety of hive systems, organic acid applications and not least the attitude of the beekeeper who performs the practical part of the application, these aspects must be taken into account. Yet, further scientific input is equally important to be collected. Exemplary for this need is a recent study that provides empirical data on how many mites a single drone frame can carry and how this is affected by the season (Odemer et al., 2022). These are important and necessary baseline data for the further development of BEEHAVE, and we very much encourage their publication.

## 5 CONCLUSION

We introduce a novel module for the BEEHAVE model, where varroa control was implemented as part of Good Beekeeping Practice. This module serves two main purposes: (1) using the model as a demonstration tool for beekeeper education and (2) providing an accessible implementation of varroa control that operates with organic acids. For the first purpose, additional data is needed on the number of drone cells that are not located on drone frames and on the seasonal development of the mite population. Such data would help us calibrate our models. The latter purpose can already be made use of, as it demonstrates the varroa control approach in principle. With varroa still the greatest threat to the Western honey bee, implementation of this module will contribute to developing new educational tools for beekeepers. It can provide valuable insights into colony and mite population dynamics to promote awareness of the rapid growth of this pest and how to effectively counter it.

## AUTHOR CONTRIBUTIONS

**Isabel Schödl:** Conceptualization (equal); data curation (equal); formal analysis (equal); methodology (equal); software (lead); writing – original draft (lead); writing – review and editing (supporting). **Richard Odemer:** Conceptualization (equal); formal analysis (supporting); funding acquisition (equal); methodology (equal); supervision (supporting); writing – original draft (supporting); writing – review and editing (equal). **Matthias A Becher:** Software (equal); supervision (supporting); writing – original draft (supporting); writing – review and editing (supporting). **Stefan Berg:** Conceptualization (supporting); supervision (supporting); writing – original draft (supporting); writing – review and editing (supporting). **Christoph Otten:** Conceptualization (supporting); supervision (supporting); writing – original draft (supporting); writing – review and editing (supporting). **Volker Grimm:** Conceptualization (equal); data curation (equal); formal analysis (equal); funding acquisition (lead); methodology (equal); software (equal); supervision (equal); writing – original draft (equal); writing – review and editing (equal). **Juergen Groeneveld:** Conceptualization (equal); data curation (supporting); formal analysis (equal); methodology (equal); software (equal); supervision (lead); writing – original draft (equal); writing – review and editing (lead).

## CONFLICT OF INTEREST

None declared.

## Data Availability

Data are available at: https://www.comses.net/codebases/f2b74150‐4911‐4c09‐8bbf‐3062c6d24e02/releases/1.2.0.

## Endnotes

^1^

https://www.varroa‐app.de/#openModal

^2^

https://www.bienenkunde.rlp.de/Bienenkunde/Varroawetter/bundesweit‐n‐PLZ
